# Utility of Surrogate Markers for Insulin Resistance: Homeostasis Model Assessment for Insulin Resistance (HOMA-IR) and Triglyceride–Glucose (TyG) Index in Identifying Metabolic Syndrome Among Adults from Resource-limited Settings

**DOI:** 10.17925/EE.2026.22.1.1

**Published:** 2026-02-05

**Authors:** Ruchita Chauhan, Shilpa Jain, Neeta Malukar, Jaya Pathak, Maitri Ajmera, Shubham Mishra, Madhur Verma

**Affiliations:** 1. Department of Biochemistry, Medical College Baroda, Vadodara, Gujarat, India; 2. Department of Biochemistry, All India Institute of Medical Sciences, Bathinda, Punjab, India; 3. R. No 5123, Central clinical laboratory, First floor, D-block, Hospital building, All India Institute of Medical Science, Bhatinda-151001, Punjab, India; 4. Department of Medicine, Medical College Baroda, Vadodara, Gujarat, India; 5. Department of Community and Family Medicine, All India Institute of Medical Sciences, Bathinda, Punjab, India

**Keywords:** Anthromopometry, clinical decision-making, clinical laboratory techniques, Homeostasis Model Assessment for Insulin Resistance (HOMA-IR), insulin resistance, metabolic syndrome, triglyceride–glucose (TyG) index

## Abstract

**Background:**

Insulin resistance (IR) is a central feature of metabolic syndrome (MetS). While the homeostasis model assessment for insulin resistance (HOMA-IR) is widely used, it has limitations in resource-constrained settings. The triglyceride–glucose (TyG) index offers a simpler alternative, but comparative data from Indian adults are limited. This study aimed to evaluate and compare the performance of the two indices as surrogate markers for IR among adults.

**Methods:**

A hospital-based cross-sectional study was conducted among 312 adults (217 without MetS and 95 with MetS), selected using stratified random sampling. Participants underwent standardized anthropometric measurements and fasting biochemical tests. MetS was defined according to the International Diabetes Federation (IDF) criteria. HOMA-IR and TyG indices were calculated and compared across demographic and clinical subgroups. Diagnostic performance was assessed using receiver operating characteristic (ROC) curve analysis.

**Results:**

Participants with MetS had significantly higher HOMA-IR and TyG index values compared with those without MetS (p<0.001). Both indices showed a strong positive correlation with metabolic parameters. ROC analysis revealed that the TyG index had superior diagnostic performance (area under the curve [AUC]=0.85) compared with HOMA-IR (AUC=0.81). The TyG index also showed greater sensitivity (68.4% versus 64.2%), specificity (94.0% versus 89.4%) and overall accuracy (86.2% versus 81.7%).

**Conclusions:**

Both HOMA-IR and the TyG indices are useful in identifying IR; however, the latter demonstrated better overall diagnostic accuracy and greater practicality. Given its ease of calculation and cost-effectiveness, the TyG index may be a suitable screening tool for IR in low-resource environments. Further longitudinal validation studies are warranted.

Insulin resistance (IR), a hallmark of various metabolic disorders, represents a diminished responsiveness of target tissues to the actions of insulin, resulting in impaired glucose uptake, dyslipidaemia and endothelial dysfunction.^1,2^ Worldwide, the prevalence of IR among adults ranges from 15.5% to 46.5%.^3–5^ The aetiology of IR is multi-factorial, involving a complex interplay of multiple factors. Broadly, genetic predisposition, environmental factors and unhealthy lifestyle, with increased consumption of refined carbohydrates, substance abuse and limited physical activity, lead to obesity, hypertension, dyslipidaemia and IR; specifically, adipose tissue dysfunction, characterized by adipocyte hypertrophy, inflammation and altered adipokine secretion, plays a critical role in IR pathogenesis.^6,7^ Additionally, the abnormal ectopic lipid accumulation affects organs such as the liver, skeletal muscle and pancreas, exacerbating metabolic dysfunction.^6^

Chronic IR is intricately linked to the pathogenesis of metabolic syndrome (MetS). MetS, a cluster of metabolic abnormalities, including abdominal obesity, dyslipidaemia (increased triglycerides and low high-density lipoprotein (HDL) cholesterol), hypertension and impaired fasting glucose, stands as a precursor to both cardiovascular disease (CVD) and type 2 diabetes mellitus (T2DM).^6,8^ Therefore, it is essential to recognize IR in its early stages, which helps prevent the onset of MetS through timely, appropriate preventive and therapeutic measures, and progression to DM and CVD can be halted or delayed. For early diagnosis of this condition, there is a need to establish markers that can help to recognize IR and MetS at an early stage.

Several expert groups have proposed clinical criteria for diagnosing MetS. The WHO (1998) first introduced the concept, emphasizing IR as a mandatory component^6^. Later, the National Cholesterol Education Program Adult Treatment Panel III (NCEP/ATP III, 2001) provided simpler clinical criteria that were more feasible for large-scale screening but did not account for ethnic variations^6^. In contrast, the International Diabetes Federation (IDF, 2005) harmonized earlier definitions by including ethnicity- and gender-specific waist circumference cut-offs, recognizing central obesity as a core feature, and offering greater applicability across diverse populations.^6,9^

Within this endeavour, current IR research focuses on improving our understanding of its molecular mechanisms, identifying novel biomarkers for early detection and developing targeted therapeutic interventions.^2,6,10^ However, there is still no universally acceptable marker for IR. Available tests for measuring IR include the insulin tolerance test, insulin suppression test, continuous infusion of glucose with model assessment, frequently sampled intravenous glucose tolerance test, oral glucose tolerance test, meal tolerance test, rapid insulin sensitivity test and the hyperinsulinaemic-euglycaemic clamp test, which is considered the gold standard, but is less feasible in routine clinical settings. Promising surrogate indices for IR include the homeostasis model assessment for insulin resistance (HOMA-IR), triglyceride–glucose (TyG) index, quantitative insulin sensitivity check index, insulin sensitivity index, oral glucose insulin sensitivity and rapid insulin sensitivity index, of which the first two are the most frequently studied markers.^11,12^ Despite HOMA-IR gaining widespread acceptance and utilization in both clinical and research settings, it is limited by its cost, accessibility, the non-feasibility for mass screening and that there is no universally accepted cut-off value for HOMA-IR for diagnosing IR.^12,13^

The TyG index has also garnered increasing attention as a surrogate measure of IR and metabolic health.^14^ It is a simple, accessible and more affordable marker than other IR measurements.^12,13^ Despite their popularity, there is a lack of consensus about the cut-off values of the HOMA-IR and TyG index for IR diagnosis in the Indian population. There are very few studies from India that have reported the cut-off levels of HOMA-IR in the adult population and their relation with the TyG index.^1,2^ With this background, the present study aims to establish HOMA-IR and the TyG index as markers of IR in the adult population. The TyG index can serve as a cost-effective and easy-to-perform screening test for IR, thereby helping to predict the risk of DM and CVD.

## Methods

### Study design

A cross-sectional study was conducted over a 6-month period, from August 2023 to January 2024.

### Study settings

This hospital-based study was carried out in the out-patient department (OPD) sample collection centre run by the Department of Biochemistry, in close collaboration with the Department of Medicine, Sir Sayajirao General Hospital (SSGH), and Medical College Baroda, Vadodara, located in Gujarat, a western state of India.

### Study population

All adults aged more than 20 years without any known illness, purposefully visiting the study area to get a laboratory test, a medical fitness certificate or routine screening, were included in this study. They were given the option to participate in the study after being informed about its details. All individuals who provided informed consent were included in the study, except those who met the exclusion criteria. The exclusion criteria included a positive history of alcoholism and participants with conditions or medications influencing glucose or lipid metabolism, including thyroid disorders, liver disease, pancreatitis, diabetes mellitus on treatment, familial hyperlipidaemias, CVD or lipid-lowering therapy, to minimize biochemical confounding.

### Sample size and sampling technique

The sample size was calculated using the online tool MedCalc, based on the ability to detect a statistically significant difference in the area under the receiver operating characteristic (ROC) curve for two indices in identifying IR.^15^ Considering an area under the curve (AUC) of 0.80 for the TyG index and 0.70 for HOMA-IR, as per previous studies, with a power of 80%, an alpha level of 0.05 and an allocation ratio of 2:1 (non-MetS:MetS), a minimum of 282 participants (188 without MetS and 94 with MetS) was estimated to be sufficient to detect this difference.^16,17^

A total of 748 adults aged ≥20 years who visited the study site between August 2023 and January 2024 were screened for participation. After obtaining informed consent, all participants underwent standardized anthropometric and biochemical assessments, including fasting plasma glucose, triglycerides, HDL cholesterol and insulin estimation. Individuals were classified as having or not having MetS according to the IDF 2005 criteria that include central obesity (waist circumference >90 cm for South and East Asian men or >80 cm for women, or body mass index [BMI] >30 kg/m²) plus any two of the following: elevated blood pressure (>130/85 mmHg), triglycerides >150 mg/dL, reduced HDL cholesterol (<40 mg/dL for men or <50 mg/dL for women) or fasting blood glucose >100 mg/dL.^9^ Given the marked ethnic variability in body composition and cardiometabolic risk among Asian Indians, the IDF criteria were considered most appropriate for this population, as they incorporate lower waist circumference thresholds and align with regional evidence on obesity and metabolic risk. Moreover, these criteria are widely used in Indian epidemiological and clinical studies, enabling comparability across research contexts. While other definitions, such as NCEP/ATP III and WHO, have conceptual and methodological value, the IDF framework provides a pragmatic and globally accepted diagnostic approach suitable for the study population.

Following classification, stratified random sampling was undertaken. Within each stratum (MetS and non-MetS), participants were assigned serial numbers, and a computer-generated random-number list (using the Random. org algorithm) was employed to select individuals in a 2:1 ratio (non-MetS:MetS).^18^ This ratio was chosen to reflect the approximate prevalence of MetS in the screened cohort and to enhance comparative power between groups. From the eligible pool, 250 participants without MetS and 100 with MetS were initially selected. After excluding incomplete records, the final analytical sample comprised 312 adults (217 non-MetS and 95 MetS), while ensuring balanced representation of both metabolic profiles and minimizing selection bias.

### Study variables

#### Dependent variables

HOMA index and TyG index were our primary dependent variables. The HOMA index was calculated using the following formula: fasting insulin (μU/mL) multiplied by fasting plasma glucose (mg/dL) and then divided by 405.^11^ Similarly, the TyG index was calculated as the natural logarithm of the product of fasting triglycerides and fasting glucose, divided by 2 – specifically, Ln (fasting triglycerides [mg/dL] × fasting glucose [mg/dL])/2.^12^

#### Independent variables

The independent variables in this study were based on a literature review and the scope of data collection. We included demographic factors (age and gender), anthropometric measurements (BMI, waist circumference, weight and height) and clinical conditions such as hypertension and diabetes status. Biochemical parameters, namely fasting plasma glucose, serum insulin, triglycerides, HDL cholesterol, total cholesterol, hemoglobin A1c (HbA1c), serum urea, creatinine, serum glutamic oxaloacetic transaminase (SGOT), serum glutamic pyruvic transaminase (SGPT) and alkaline phosphatase (ALP), were also considered.

#### Study protocol

Eligible participants reporting to the out-patient laboratory sample collection centre for medical fitness or routine screening were first approached by the research team. The study’s purpose and procedures were explained in detail, and written informed consent was obtained from each individual before enrolment. Only those who voluntarily consented and met the eligibility criteria were included in the study. Following consent, participants underwent anthropometric and physiological assessments performed by trained staff using standardized methods. Weight and height were measured using calibrated instruments, and waist circumference and blood pressure were measured using standard protocol.^19^ BMI was categorized using the Asian Indian criteria for BMI classification.^6^

Venous blood samples were collected after confirming an overnight fasting state. Appropriate vacutainers were used for different biochemical analyses: plain vacutainers for lipid profile, insulin, renal and liver function tests; fluoride vacutainers for plasma glucose; and ethylenediaminetetraacetic acid (EDTA) vacutainers for HbA1c and haematological parameters. All the routine biochemical tests were performed on the ERBA XL-640 fully automated biochemistry analyser (Transasia Bio-Medicals Ltd., Mumbai, India). Serum insulin was measured on an Abbott Architect i-1000 SR fully automated chemiluminescent immunoassay analyser (Abbott Laboratories Diagnostics division, Abbott Park, IL, USA). All biochemical parameters were measured using standard enzymatic or kinetic methods.

#### Statistical analysis

Statistical analysis was conducted using MedCalc software. The distribution of continuous variables was assessed using the Shapiro–Wilk test. Variables following a normal distribution were summarized as mean ± standard deviation, and group comparisons were performed using the unpaired t-test. Variables that were not normally distributed were expressed as median with interquartile range and compared using the Mann–Whitney U test. Categorical variables were presented as frequencies and percentages, and differences between groups were evaluated using the chi-square test. Correlation between key continuous indices was assessed using Spearman’s rank correlation coefficient. ROC curve analysis was carried out to evaluate the discriminatory performance of the indices in predicting IR. Optimal cut-off points were determined using the Youden index (J = sensitivity + specificity - 1) to maximize combined diagnostic performance. AUC 0.71–0.80 depicts fair, 0.81–0.90 depicts good and 0.91–1.00 depicts excellent diagnostic performance. For each index, the AUC, sensitivity, specificity, positive predictive value (PPV) and negative predictive value (NPV), accuracy and optimal cut-off points were reported. All statistical tests were two-tailed, and a p-value of <0.05 was considered statistically significant.

#### Ethics statement

Ethical clearance for the study was obtained from the Institutional Ethics Committee before data collection. Written informed consent was obtained from all participants after they were informed about the purpose, procedures and voluntary nature of their participation. Confidentiality of personal and medical information was strictly maintained throughout the study. All procedures involving human participants were conducted in accordance with the ethical standards of the institutional committee and the 1964 Helsinki Declaration and its subsequent amendments, or with comparable ethical standards.

## Results

The mean age of the 312 participants was 40.4 ± 13.2 years (range: 20–80 years). Group I (without MetS, n=217) had a mean age of 38.1 ± 12.8 years, while group II (with MetS, n=95) had a significantly higher mean age of 45.7 ± 12.5 years (p<0.0001). Females (52.2%) slightly outnumbered males (47.8%) overall, with a similar gender distribution across both groups. *Table 1* summarizes the comparison between participants with MetS (n=95) and those without MetS (n=217). The gender distribution was similar across groups (p=0.560). However, participants with MetS were significantly older, with 47.4% being over 45 years old compared with 24.4% in the non-MetS group (p<0.001). Obesity markers, including BMI, weight and waist circumference, were significantly higher in the MetS group for both sexes (p<0.001). Hypertension and diabetes were also more prevalent among those with MetS (42.1% versus 1.8% and 35.8% versus 0.9%, respectively; p<0.001). Blood pressure readings were significantly elevated in the MetS group (systolic blood pressure [SBP]: 130 versus 120 mmHg; diastolic blood pressure [DBP]: 86 versus 78 mmHg; p<0.001). Fasting glucose, serum insulin, HbA1c and triglycerides were all significantly higher in the MetS group (p<0.001), while HDL cholesterol was lower (p=0.002). ALP levels were also elevated in participants with MetS (p=0.004). No significant differences were observed in total cholesterol, urea, creatinine, SGOT or SGPT.

*Table 2* highlights significant differences in the HOMA-IR index between individuals with and without MetS across various demographic and clinical subgroups. In all strata, HOMA-IR values were consistently and significantly higher among those with MetS (p<0.05). By gender, males with MetS had a median HOMA-IR of 4.2 compared with 1.5 in those without MetS (p<0.001), while females showed a similar pattern (3.2 versus 1.6; p<0.001). HOMA-IR values increased with age, and in all age categories, individuals with MetS had significantly higher HOMA-IR values than those without MetS. Obesity status strongly influenced HOMA-IR: overweight or obese individuals with MetS had a median HOMA-IR of 3.4, compared with 1.7 in non-MetS individuals (p<0.001). Hypertensive individuals with MetS had marginally higher HOMA-IR compared with their non-MetS counterparts (p=0.050), while among non-hypertensive participants, the difference was more pronounced (p<0.001). Participants with fasting hyperglycaemia exhibited higher HOMA-IR values overall, particularly within the MetS group (7.3 versus 1.1; p=0.003).

**Table 1:** Demographic and clinical characteristics of the participants with or without metabolic syndrome

Demographic and clinical parameters	With metabolic syndrome No. (col%) 95 (100)	Without metabolic syndrome No. (col%) 217 (100)	p-value
Total			
**Gender**			0.560
Male	43 (45.3)	106 (48.8)	
Female	52 (54.7)	111 (51.2)	
**Age (completed years)**			<0.001
<30	9 (9.5)	65 (30.0)	
30–45	41 (43.2)	99 (45.6)	
>45	45 (47.4)	53 (24.4)	
**Body mass Index**			<0.001
Underweight or normal	95 (43.8)	10 (10.5)	
Overweight or obese	122 (56.2)	85 (89.5)	
Among males	28.5 ± 5.7	23.2 ± 2.8	<0.001
Among females	28.9 ± 4.2	24.0 ± 3.3	<0.001
**Hypertension**			<0.001
Present	40 (42.1)	4 (1.8)	
Absent	55 (57.9)	213 (98.2)	
Systolic blood pressure (mmHg)	130 (116–146)	120 (110–122)	<0.001
Diastolic blood pressure (mmHg)	86 (76–90)	78 (70–80)	<0.001
**Fasting hyperglycaemia**			<0.001
Present	34 (35.8)	2 (0.9)	
Absent	61 (64.2)	215 (99.1)	
Plasma fasting glucose (mg/dL)	110 (101–144)	89 (82–94)	<0.001
Serum insulin (uU/mL)	12.1 (7.3–18.5)	7.2 (4.9–9.5)	<0.001
Glycosylated haemoglobin – HbA1c (%)	5.4 (4.9–5.8)	4.9 (4.4–5.2)	<0.001
Anthropometry*			
**Height**			
Male	1.7 ± 0.1	1.7 ± 0.1	0.926
Female	1.6 ± 0.1	1.6 ± 0.1	0.171
**Weight**			
Male	78.3 ± 18.3	63.8 ± 11.3	<0.001
Female	74.2 ± 13.8	60.2 ± 10.1	<0.001
**Waist circumference (cm)**			
Male	40.2 ± 4.9	32.9 ± 2.0	<0.001
Female	39.3 ± 4.4	29.9 ± 1.2	<0.001
**Other biochemical profile†**			
Triglyceride (mg/dL)	157 (104–191)	99 (77–120)	<0.001
Cholesterol (mg/dL)	182 (136–214)	169 (138–189)	0.442
High-density lipoprotein (mg/dL)	51 (38–60)	52 (50–57)	0.002
Urea (mg/dL)	23 (18–30)	22 (17–27)	0.852
Creatinine (mg/dL)	0.86 (0.72–1.02)	0.85 (0.71–0.98)	0.171
Serum glutamic-oxaloacetic transaminase (IU/L)	26 (22–34)	27 (20–36)	0.419
Serum glutamic pyruvic transaminase (IU/L)	24 (17–35)	23 (15–32)	0.055
Alkaline phosphatase (IU/L)	99 (81–135)	87 (70–106)	0.004

*Presented as mean ± SD

*†Depicted using median (IQR)*

*IQR = interquartile range; SD = standard deviation.*

**Table 2:** Comparison of homeostasis model assessment index between individuals with and without metabolic syndrome as per demographic and clinical profile

Sociodemographic and clinical profile	Overall	With metabolic syndrome	Without metabolic syndrome	p-value
**Gender**				
Male	1.7 (1.2–2.7)	4.2 (2.3–7.2)	1.5 (1–1.9)	<0.001
Female	1.8 (1.2–2.8)	3.2 (1.6–5.6)	1.6 (1–2.2)	<0.001
**Age (completed years)**				
<30	1.7 (1–2.4)	3.3 (3.1–5.1)	1.5 (1–2.2)	<0.001
30–45	1.7 (1.2–2.8)	3.3 (1.7–6.9)	1.5 (1.1–2)	<0.001
>45	1.9 (1.3–3.5)	3.5 (2–6.5)	1.7 (1–1.9)	<0.001
**Body mass index**				
Underweight or normal	2 (1.4–3.3)	3.4 (2–6.9)	1.7 (1.2–2.3)	<0.001
Overweight or obese	1.4 (0.9–2)	3.5 (1.6–4.6)	1.3 (0.8–1.9)	0.011
**Hypertension**				
Present	2.7 (1.6–5)	3.1 (1.7–5.6)	0.9 (0.6–2.7)	0.050
Absent	1.7 (1.2–2.6)	3.8 (2.3–7.2)	1.6 (1–2.1)	<0.001
**Fasting hyperglycaemia**				
Present	7.3 (3–8.7)	7.3 (3.2–8.8)	1.1 (0.7–1.6)	0.003
Absent	1.7 (1.1–2.5)	2.7 (1.6–4.4)	1.6 (1–2.1)	<0.001

**Figure 1:**
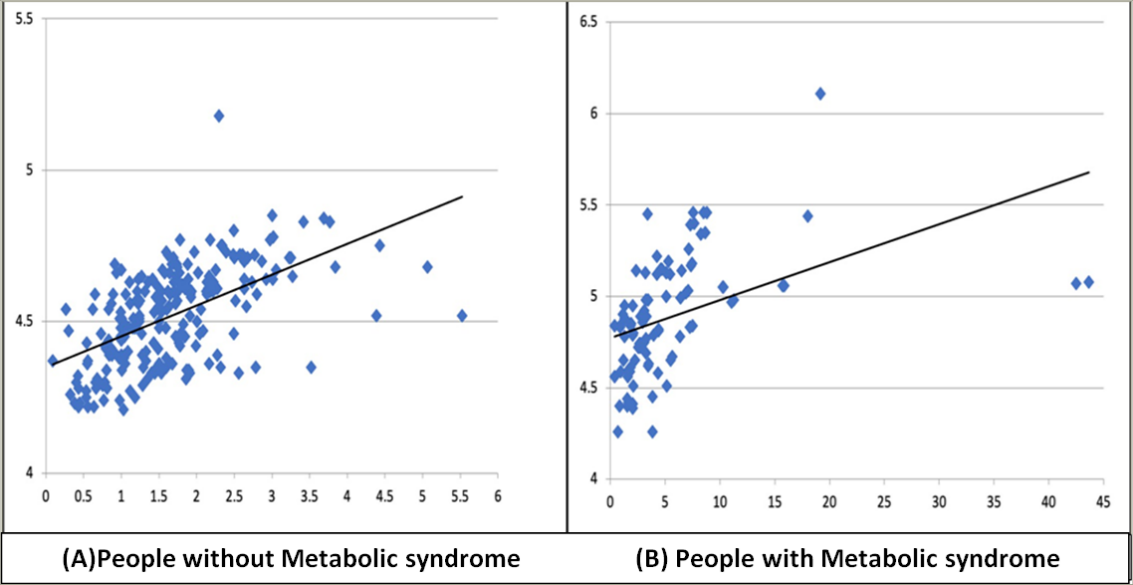
Correlation of homeostasis model assessment index and triglyceride–glucose index in people with and without metabolic syndrome

Likewise, *Table 3* shows a consistent elevation of the TyG index among individuals with MetS across all examined subgroups. The differences were statistically significant (p<0.05) in each category. The index was significantly higher among both males and females with MetS compared with their non-MetS counterparts (males: 4.9 versus 4.6; females: 4.8 versus 4.5; both p<0.001). A similar pattern was observed across all age groups, with TyG values significantly higher among those with MetS, even in the <30 years age group (4.8 versus 4.5; p=0.008). The TyG index depicted higher values among adults living with overweight or obesity and MetS (4.9 versus 4.6; p<0.001). Even among participants with underweight or normal BMI, the difference remained significant (4.7 versus 4.5; p=0.022). The index was also significantly elevated in participants with hypertension or incidental hyperglycaemia, particularly in hypertensives (4.8 versus 4.4; p=0.002) than in diabetics (5.1 versus 4.6; p=0.013). Among those without these conditions, the TyG index still discriminated effectively between MetS and non-MetS groups, depicting consistent patterns across sex, age and BMI categories, demonstrating its robustness across diverse demographic and clinical strata. A significant positive correlation was observed between the HOMA-IR and TyG index in both participants without MetS (r=0.605) and those with MetS (r=0.65), as shown in *Figure 1A, B*, indicating a strong alignment between the two indices across metabolic states.

**Figure 2:**
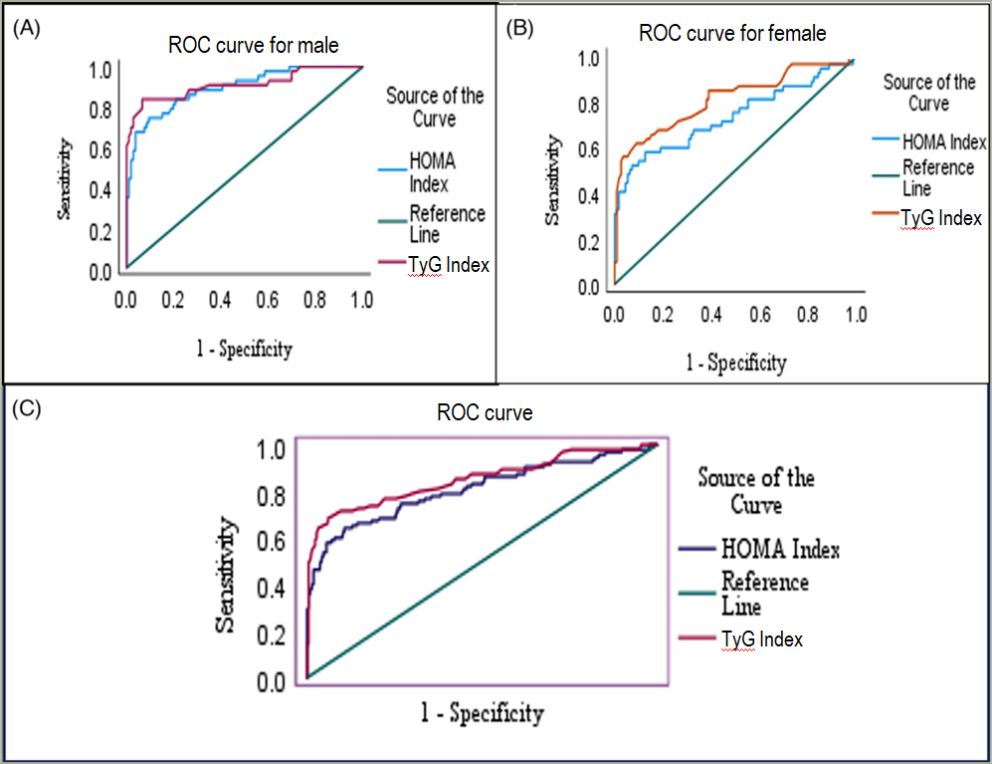
Diagnostic performance of homeostasis model assessment for insulin resistance and triglyceride–glucose indices for detecting insulin resistance

*Table 4* compares the discriminatory performance of the HOMA-IR and TyG indices in identifying IR using ROC analysis. Overall, the TyG index outperformed the HOMA-IR index across all metrics. The TyG index demonstrated a higher AUC (0.85 versus 0.81), with superior sensitivity (68.4% versus 64.2%), specificity (94.0% versus 89.4%) and overall accuracy (86.2% versus 81.7%). Its PPV and NPV were also higher (PPV: 83.3% versus 72.6%; NPV: 87.2% versus 85.1%). Gender-stratified analysis further confirmed the robustness of the TyG index. Among males, the TyG index showed an AUC of 0.91 and accuracy of 90.6%, compared with HOMA-IR’s AUC of 0.89 and accuracy of 85.9%. In females, the TyG index again outperformed HOMA-IR (AUC: 0.81 versus 0.74; accuracy: 81.6% versus 77.9%). The optimal cut-off for IR was 2.67 for the HOMA-IR and 4.73 for the TyG index. *Figure 2A–C* presents the ROC curves comparing the diagnostic performance of HOMA-IR and the TyG index in identifying IR, stratified by sex (*Figure 2A, B* for males and females, respectively) and for the overall population (*Figure 2C*). The graphical findings supplement the tabulated ROC metrics. Overall, the TyG index consistently demonstrated superior discriminatory ability, with higher AUC values, especially in males, but with a less pronounced pattern in females (*Figure 2A–C*).

**Table 3:** Comparison of triglyceride–glucose index between individuals with and without metabolic syndrome as per sociodemographic and clinical profile

Sociodemographic and clinical profile	Overall	With metabolic syndrome	Without metabolic syndrome	p-value
**Gender**				
Male	4.6 (4.5–4.8)	4.9 (4.8–5.2)	4.6 (4.4–4.7)	<0.001
Female	4.6 (4.4–4.7)	4.8 (4.6–5.1)	4.5 (4.4–4.6)	<0.001
**Age (completed years)**				
<30	4.5 (4.4–4.6)	4.8 (4.6–4.9)	4.5 (4.4–4.6)	0.008
30–45	4.6 (4.5–4.7)	4.9 (4.7–5.1)	4.6 (4.4–4.7)	<0.001
>45	4.6 (4.5–4.8)	4.8 (4.6–5.1)	4.5 (4.4–4.6)	<0.001
**Body mass index**				
Underweight or normal	4.6 (4.5–4.8)	4.9 (4.7–5.1)	4.6 (4.5–4.6)	<0.001
Overweight or obese	4.5 (4.4–4.6)	4.7 (4.4–5.2)	4.5 (4.3–4.6)	0.022
**Hypertension**				
Present	4.7 (4.5–5)	4.8 (4.6–5)	4.4 (4.3–4.4)	0.002
Absent	4.6 (4.4–4.7)	4.9 (4.8–5.1)	4.5 (4.4–4.6)	<0.001
**Fasting hyperglycaemia**				
Present	5.1 (4.8–5.3)	5.1 (4.8–5.3)	4.6 (4.6–4.7)	0.013
Absent	4.6 (4.4–4.7)	4.8 (4.6–5)	4.5 (4.4–4.6)	<0.001

*A, B: Correlation of homeostasis model assessment index (X aixs) and triglyceride–glucose index (Y axis) in people without (A) and with metabolic syndrome (B) HOMA = homeostasis model assessment; TyG = triglyceride–glucose.*

## Discussion

MetS and IR are intricate components of cardiometabolic risk, yet identifying practical and scalable markers for early detection remains a public health challenge. In this context, our study offers meaningful insights by comparing two widely studied surrogate markers (HOMA-IR and TyG index) among adults with and without MetS in a hospital-based setting. We observed certain intriguing clinical findings. First, adults with MetS exhibited significantly higher levels of IR markers, along with elevated cardiometabolic risk factors, including BMI, blood pressure, triglycerides and fasting glucose. In addition to central obesity, the second most common feature was high plasma glucose. Second, both indices showed strong and consistent discriminatory performance within our study population across all demographic and clinical subgroups, including age, sex, BMI, diabetes and hypertension status. Third, the TyG index demonstrated a strong positive correlation with HOMA-IR and consistently outperformed it in ROC analysis, with a higher AUC and overall accuracy. Finally, the superior diagnostic performance of the TyG index was particularly evident in males, although it remained a reliable marker across both sexes.

**Table 4:** Receiver operating characteristic curve depicting the discriminatory performance of indices for insulin resistance (homeostasis model assessment index and triglyceride–glucose index)

Parameter	AUC (95% CI)	Sensitivity (%)	Specificity (%)	PPV (%)	NPV (%)	Accuracy	Cut-off
**HOMA index**							
Overall	0.81 (0.75–0.87)	64.2	89.4	72.6	85.1	81.7	2.6756
Male	0.89 (0.84–0.95)	74.4	90.6	76.2	89.7	85.9	2.5681
Female	0.74 (0.64–0.83)	57.7	87.4	68.2	81.5	77.9	2.6756
**TyG index**							
Overall	0.85 (0.80–0.90)	68.4	94.0	83.3	87.2	86.2	4.7350
Male	0.91 (0.85–0.97)	83.7	93.4	83.7	93.4	90.6	4.7350
Female	0.81 (0.73–0.89)	61.5	91.0	76.2	83.5	81.6	4.7150

*AUC = area under the curve; CI = confidence interval; HOMA = homeostasis model assessment; NPV = negative predictive value; PPV = positive predictive value; TyG = triglyceride–glucose.*

*A: ROC curve for male participants showing the AUC for HOMA-IR and TyG indices; B: ROC curve for female participants; C: ROC curve for the overall study population. The diagonal green line represents the reference line (AUC=0.5), indicating no discrimination. Values closer to the top-left corner indicate better sensitivity and specificity. AUC = area under the curve; HOMA-IR = homeostasis model assessment for insulin resistance; ROC = receiver operating characteristic; TyG = triglyceride–glucose.*

In our study, the maximum number of individuals with MetS was in the middle-age group (41–50 years), a finding in agreement with earlier studies.^20–26^ Our study’s finding that adults with MetS had significantly higher levels of IR markers (HOMA-IR and TyG index) aligns with the most widely accepted and unifying hypothesis describing the pathophysiology of MetS, according to which the IR, which is defined as a defect in insulin action, leads to fasting hyperinsulinaemia to maintain normal blood sugar levels.^7^ HOMA-IR was first introduced in 1985 to evaluate β-cell function and IR by analysing basal glucose and insulin.^27^ Insulin concentration is dependent on β-cell response to glucose, which, in turn, signals glucose uptake in adipose tissue and muscles. The equation for HOMA-IR uses fasting serum insulin concentration in the numerator and fasting plasma glucose concentration in the denominator, so its higher level means glucose level is higher despite sufficient insulin, indicating IR.

The TyG index has also emerged as a valuable parameter in recent medical laboratory research focusing on IR-related disorders and CVD.^28,29^ Its calculation is straightforward and cost-effective, relying solely on fasting blood triglyceride and glucose measurements, which are standard procedures in hospitals and clinical laboratories.^30^ The biochemical basis for using the TyG index as a marker of IR is that an overabundance of circulating fatty acids is both a cause and an effect of IR. The most sensitive pathway of insulin action is the inhibition of lipolysis in adipose tissue.^31^ Therefore, when IR develops, the increased lipolysis of stored triacylglycerol molecules in adipose tissue produces more fatty acids, which could further inhibit the antilipolytic effect of insulin, leading to additional lipolysis.^7^ Enhanced lipolysis in adipose tissues leads to increased circulating free fatty acids, which, in turn, result in increased hepatic production of glucose, triglycerides and the secretion of very low-density lipoproteins. Decreased insulin sensitivity in muscle inhibits insulin-mediated glucose uptake, leading to reduced glucose storage as glycogen and increased lipid accumulation as triglyceride (TG). The TyG index integrates fasting triglyceride and glucose levels, capturing the interplay between hyperglycaemia and hypertriglyceridaemia, both of which result from impaired insulin action in hepatic and peripheral tissues and thus underpins its utility in evaluating IR.

We observed that adults with MetS had significantly higher levels of IR markers, along with elevated cardiometabolic risk factors such as BMI, blood pressure, triglycerides and fasting glucose, in agreement with Zheng et al.^32^ We found the best cut-off value for the HOMA index to predict IR was 2.6, with a sensitivity of 65.26%, specificity of 86.64%, PPV of 68.13% and NPV of 85.07%. Aman et al. reported a cut-off for the HOMA index of IR >2.24.^12^ Salazar et al. reported an HOMA index cut-off of >2.^16^ Zheng et al. reported an HOMA-IR cut-off of >2.5.^32^ We found the best cut-off value for the TyG index to predict IR was 4.73, with a sensitivity of 69.47%, specificity of 92.63%, PPV of 80.49% and NPV of 87.39%. Aman et al. reported that the cut-off value for the TyG index was 4.66, with a sensitivity of 86.2%, rather than a low specificity of 44.1%.^12^ Salazar et al. reported a single cut-off point of 4.5 for the TyG index to classify individuals with IR among 2,230 study subjects aged >18 years.^16^ Guerrero-Romero et al. found that the TyG index showed a strong association with the hyperinsulinemic euglycemic clamp (HEC) method in diagnosing IR (AUC: 0.858).^32^ A TyG index cut-off of 4.68 exhibited a sensitivity of 96.5% and a specificity of 85% for estimating IR in adults.^33^ The cut-off values derived in our study (HOMA-IR ≥2.6; TyG ≥4.73) are clinically meaningful within this sample, as they align closely with previously reported thresholds and provide a balance between sensitivity and specificity, as determined by the Youden index.^34^ These cut-offs can aid early detection of IR and demonstrate the TyG index’s potential for inclusion in routine laboratory screening, especially in low-resource settings.

We observed high discriminatory power of both indices across all demographic and clinical subgroups. The TyG index had a strong positive correlation with the HOMA index in group I (ρ=0.605), group II (ρ=0.650) and the combined group (ρ=0.685). The TyG index showed higher sensitivity (69.47% versus 65.26%), specificity (92.63% versus 86.64%) and overall accuracy (87.39% versus 85.07%) for predicting IR. Of the two indices, the TyG index consistently outperformed it in ROC analysis, with higher AUC and overall accuracy to determine the occurrence of MetS, which is in agreement with previous studies.^12,16,35,36^ Moon et al. and Simental-Mendía et al. reported that the TyG index shows a stronger correlation with several obesity-related factors, including BMI, fasting insulin and lipoproteins, compared with HOMA-IR.^14,37^ Our findings align with recent evidence syntheses showing that the TyG index has good diagnostic performance for IR and meaningful prognostic value across outcomes. A systematic review confirmed TyG’s accuracy for identifying IR in clinical and community settings, while meta-analyses link higher TyG to incident T2DM in middle-aged adults, microvascular complications such as diabetic retinopathy, and macrovascular endpoints including cerebrovascular disease and major adverse cardiovascular events (particularly among patients with hypertension).^28,38–41^ These reviews reinforce our stance on the use of TyG as a low-cost, risk-stratification tool that complements or, in resource-limited contexts, substitutes insulin-based indices.

While HOMA-IR remains widely used, its reliance on insulin assays limits its feasibility in routine practice. The ease of obtaining triglyceride and glucose measurements from standard blood tests makes the TyG index a simpler, more economical and practical tool for widespread screening. The screening can help to detect IR at an early stage so that timely, appropriate treatment can be started, and progression to DM and CVD can be halted or delayed. However, the superior diagnostic performance of the TyG index was particularly evident in males, although it remained a reliable marker across both sexes.

## Strengths and Limitations

There are a few strengths and limitations of this study that should be acknowledged. A major strength lies in the use of a well-equipped and quality-assured clinical biochemistry laboratory adhering to International Organization for Standardization (ISO) 15189, which ensured the reliability and accuracy of all biochemical measurements through automated analysers and standardized assay methods. This enhances the internal validity of the study and minimizes measurement bias. Additionally, there was a robust comparative evaluation of two widely used surrogate markers across a well-defined adult population, using standardized anthropometric and biochemical assessments, backed up by a robust sample size calculation. MetS was diagnosed as per the updated IDF criteria. The inclusion of both individuals with and without MetS enabled meaningful subgroup analyses and enhanced the generalizability of the findings. In addition, the consistency of findings across multiple demographic and metabolic subgroups strengthens internal validity and reproducibility of the results. To the best of our knowledge, this is the first study regarding MetS, HOMA index and TyG index in the Central Gujarat region, where the prevalence of MetS and diabetes is very high. However, this study does have some critical limitations. Being a hospital-based study with a cross-sectional design, there is a chance of selection bias, and it cannot establish causal relationships. While our sample was reasonably representative of apparently healthy adults, the hospital-based study settings may limit the generalizability of the findings to the broader community, particularly populations with different health-seeking behaviours. Furthermore, the stringent exclusion criteria used to control biochemical confounders and enhance internal validity also narrow the generalizability of the findings to real-world populations, where these metabolic disorders commonly coexist. However, the exclusion of participants with overt diabetes or CVD may have led to an underestimation of IR severity and limited applicability to typical clinical populations, where such comorbidities are common. Furthermore, the effects of diet and physical activity have not been studied, which are crucial parameters in the natural history of the MetS, and we recommend their inclusion in future longitudinal studies. Additionally, IR in our study was assessed only using surrogate indices without comparing them against a gold-standard method, such as the hyperinsulinaemic-euglycaemic clamp test. Although this test remains the most accurate measure, it is not feasible for routine use because of its complexity, time requirements and cost. Therefore, validation of these surrogate indices against clamp-based measures is desirable. This limitation should be interpreted with caution, as it may affect external validity and the precision of IR estimates. Moreover, the cut-off values proposed for HOMA-IR and TyG indices were derived from a single regional sample and may not be directly generalizable to all Indian populations; hence, external validation across diverse ethnic and regional cohorts is required before routine application. Future studies should aim to compare both indices with gold-standard techniques in representative Indian populations.

Several important clinical implications and recommendations have emerged from our study. First, our findings reinforce the value of early identification of IR in individuals with MetS, even in the absence of overt diabetes. The significant elevation of both indices across all high-risk subgroups, including those living with overweight/obesity, hypertension and impaired glucose metabolism, highlights the need for routine metabolic screening in such populations. The TyG index, given its simplicity, affordability and superior diagnostic accuracy as demonstrated in our study, presents a practical alternative to HOMA-IR, particularly in resource-constrained settings where insulin assays are either unavailable or unaffordable. Its reliance on readily available parameters, such as fasting glucose and triglycerides, makes it feasible for integration into routine primary care and screening programmes. Additionally, the consistent correlation between TyG and HOMA-IR across various metabolic states supports its utility as a surrogate for IR, not only in research settings but also for clinical risk stratification. This may help clinicians identify high-risk individuals earlier, enabling timely intervention through lifestyle modification or pharmacological management. Based on these findings, we suggest that the TyG index may serve as a useful adjunctive tool for early identification of IR, rather than a replacement for standard diagnostic protocols. Future studies should validate its predictive utility in longitudinal cohorts and evaluate its role in guiding preventive interventions to reduce cardiometabolic risk.

## Conclusion

In conclusion, this study highlights the clinical relevance of both HOMA-IR and the TyG index as surrogate markers of IR. While both indices showed strong and consistent discriminatory performance within our study population, the TyG index emerged as a more accessible and cost-effective alternative, offering superior diagnostic accuracy, particularly in male participants. Given its simplicity, affordability and strong correlation with HOMA-IR, the TyG index holds promise as a routine screening tool for early identification of IR in resource-limited and primary care settings. Incorporating it into clinical practice could facilitate timely risk stratification and preventive interventions for cardiometabolic diseases. Further prospective studies are warranted to validate its prognostic value and define standardized cut-offs tailored to the Indian population.
